# Sensing of Damage and Repair of Cement Mortar Using Electromechanical Impedance

**DOI:** 10.3390/ma12233925

**Published:** 2019-11-27

**Authors:** Hussameldin Taha, Richard J. Ball, Kevin Paine

**Affiliations:** BRE Centre for Innovative Construction Materials, Department of Architecture and Civil Engineering, University of Bath, Claverton Down BA2 7AY, UK; R.J.Ball@bath.ac.uk (R.J.B.);

**Keywords:** structural health monitoring, damage detection, electromechanical impedance, PZT, mortar

## Abstract

Lead zirconium titanate (PZT) has recently emerged as a low-cost material for non-destructive monitoring for civil structures. Despite the numerous studies employing PZT transducers for structural health monitoring, no studies have assessed the effects of both damage and repair on the electromechanical impedance response in cementitious materials. To this end, this study was conducted to assess the effects of the damage and repair of mortar samples on the electromechanical response of a surface-mounted PZT transducer. When damage was introduced to the specimen in stages, the resonance frequencies of the admittance signature were shifted to lower frequencies as the damage increased, and an increase in the peak amplitude was detected, indicating an increase in the damping and a reduction in the material stiffness properties. Also, increasing the damage in the material has been shown to decrease the sensitivity of the PZT to further damage. During the repair process, a noticeable difference between the after-damage and the after-repair admittance signatures was noted. The root-mean-square deviation (RMSD) showed a decreasing trend during the repair process, when compared to the before repair RMSD response which indicated a partial recovery for the material properties by decreasing the damping property in the material.

## 1. Introduction

Due to the increase in urbanization, and hence the number of strategic structures such as nuclear power stations, bridges, dams, and high-rise buildings, structural health monitoring (SHM) has become an important task which should be performed on a regular basis to maintain the functionality and safety during the service life-span of these structures. Ranging from catastrophic failure prevention to decreasing the running cost by detecting early damage [1,2,3,4,5,6], the acquisition and provision of a reliable and robust SHM system is crucial and should be acquired before putting the structure into service.

Despite the invaluable benefits that are obtained from deploying an efficient SHM system, SHM for massive structures represents a challenge, as traditionally, visual inspections are normally conducted only on critical structure parts where the damage is already expected to take place. Therefore, in addition to experienced inspectors and the pre-knowledge of damage locations, SHM is a laborious, labor-intensive task which involves health and safety risks, especially when it is performed manually [7]. Due to this, several SHM approaches have taken further steps towards automated/real-time sensing systems in a bid to reduce human intervention as well as to increase the safety and frequency of data collection [8,9].

In recent decades, piezo ceramics, particularly lead zirconium titanate (PZT), have been investigated as sensing materials for detecting damage in civil engineering structures. Their simplicity, low weight, economic cost, and robustness provide an ideal combination of properties for use in autonomous sensing systems. Of particular importance is their potential to provide the signal needed to activate complementary structural healing technologies such as shape memory polymers and vascular networks [10,11]. Sensing using PZT transducers is normally utilized through two main techniques, which are the active mode technique and the electromechanical impedance technique (EMI). In the active mode technique or wave propagation technique, two PZT transducers are used. One transducer acts as an actuator, which sends elastic mechanical waves through the investigated structure, and the other acts as a detector to sense the transmitted vibrational waves, in which the latter are transformed to electrical voltage signals through the direct piezoelectricity effect. Changes in the sensor voltage over time are considered as indications of changes in the material properties such as stiffness/mass [12,13,14]. Despite the successful application of this approach in sensing damage, some factors, such as the applied actuator’s voltage, the distance between the actuator and the sensor, and the frequency of actuation, require careful consideration during the monitoring phase if a noise-free signal is required [15].

The second technique is the electromechanical impedance (EMI) technique in which only one PZT transducer is used which works as both an actuator and detector simultaneously. The admittance/impedance signature of the PZT transducer is collected by an impedance analyzer over a wide frequency range. The changes in the collected signature between the pristine stage and the subsequent post-damage stages are used as indicators of the changes in the mechanical properties of the investigated structure. Due to the relatively high frequency range used in this technique, the kHz frequency range, the admittance/impedance signature caries information about the local modes and structural impedance of the surrounding material; therefore, local small-scale damage can be detected. This, in particular, is considered as an advantage for this technique, as this is in contrast to the global vibration methods, which use frequencies <100 Hz to investigate the structural conditions, which does not allow small-scale incipient damage to be detected [16,17].

### 1.1. EMI Technique Theoretical Background

**Figure 1:**
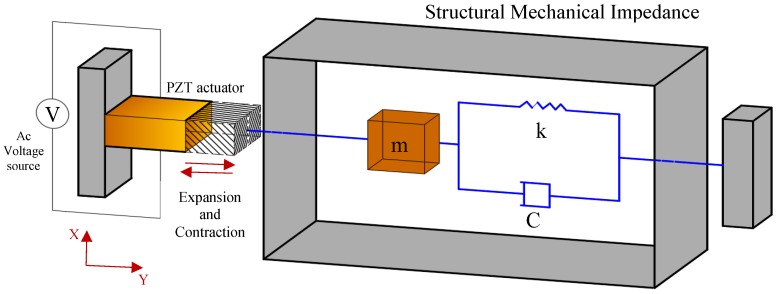
One-dimensional model for the interaction between the PZT actuator and the hosting structure.

The mechanical interaction between a PZT actuator and the host structure in the EMI method, was modeled by Liang and Sun [5]. The 1D model that they proposed assumed that the host mechanical system was a spring, mass, and damping system. The host system in this model was assumed to be driven by the lead zirconium titanate (PZT) actuator, as shown in Figure 1. 

By solving the PZT motion equation and substituting in the constitutive PZT relations which are the direct piezoelectric effect, Equation (1), and the converse effect, Equation (2), the relation between the admittance signature, the mechanical impedance of the host structure, and the mechanical impedance (SMI) of the PZT can be expressed as shown in Equation (3). Therefore, any changes in the SMI of the host structure are reflected in the admittance signature, given that the properties of the PZT actuator are maintained at a constant level.

(1) $$S_{2}=\;{\bar{S}}_{22}^{E}T_{2}+d_{32}E$$

(2) $$D_{3}=\;\varepsilon_{33}^{-T}E+d_{32}T_{2}$$

(3) $$Y=i\omega \frac{w_{A}l_{A}}{h_{A}}\left\{\frac{d_{32}^{2}{\bar{Y}}_{22}^{E}Z_{A}}{Z+Z_{A}}\frac{\tan kl_{A}}{kl_{A}}+\varepsilon_{33}^{-T}-d_{32}^{2}{\bar{Y}}_{22}^{E}\right\}$$

where $S_{2}$ is the strain in direction 2, which is the Y direction in Figure 1; ${\bar{S}}_{22}^{E}$ is the elastic compliance at zero electrical field; $d_{32}$ is the piezoelectric coefficient; $T_{2}$ is the axial stress in direction 2; $D_{3}$ is the electric displacement perpendicular to the PZT patch, which is the Z direction; *E* is the external electric field; $\varepsilon_{33}^{-T}$ is the complex electric permittivity in direction 3 at zero stress; *Y* is the complex admittance, $i=\sqrt{-1}$; ω is the excitation frequency; $w_{A},\;l_{A}\;,\;$ and $h_{A}$ are the width, length, and thickness of the PZT patch, respectively; ${\bar{Y}}_{22}^{E}$ is the complex modulus of the PZT patch at zero electrical field; *Z* and $Z_{A}$ are the mechanical impedances for the hosting structure and the PZT patch, respectively; $k=\sqrt{\frac{\omega^{2}\rho }{{\bar{Y}}_{22}^{E}}}$; and ρ is the density of the PZT patch.

Different statistical damage indexes were used to quantify the similarity between the pristine stage real part admittance signature and the subsequent post-damage stage signatures, which then were related to the changes in the host structure properties. The root-mean-square deviation (RMSD), correlation coefficient deviation (CCD), and mean absolute percentage deviation (MAPD) were all used as damage indexes in the EMI technique, see Equations (4)–(8) respectively [18,19,20]. Bhalla [18] suggested that the RMSD is the most accurate damage index.

(4) $$RMSD=\sqrt{\frac{\sum_{i=1}^{N}{\left(y_{i}-x_{i}\right)}^{2}}{\sum_{i}^{N}{\left(x_{i}\right)}^{2}}}$$

(5) CCD = 1−CC

in which

(6) $$CC=\;\frac{Cov\left(x,y\right)}{\sigma_{x}\times \sigma_{y}}$$

(7) $$Cov=\frac{1}{N}\overset{N}{\underset{i=1}{\sum }}\left(x_{i}-\bar{x}\right)\left(y_{i}-\bar{y}\right)$$

(8) $$MAPD=\frac{100}{N}\overset{N}{\underset{i=1}{\sum }}\left|\frac{y_{i}-x_{i}}{x_{i}}\right|$$

in which $x_{i}\;\mathrm{and}\;y_{i}$ are the real parts of the admittance before and after the damage, respectively. $\sigma_{x}\;\mathrm{and}\;\sigma_{y}$ are the standard deviations for the signature before and after damage. $\bar{x}$ and $\bar{y}$ are the mean values of the signature before and after the damage.

### 1.2. EMI for Previous SHM Studies

Due to the simplicity and reduced instrumentation required by the EMI technique, many studies have explored its feasibility in SHM for civil structures. In terms of concrete civil structures, Soh and Tseng [21] monitored a reinforced concrete (RC) bridge by distributing 11 PZT batches of 10 mm × 10 mm × 0.30 mm at different locations on both the compression and the tension zones of the bridge deck slab. The authors have shown that the surface-bonded PZTs were sensitive to detecting hairline cracks, which appeared in the immediate vicinity of the patches. The study did not suggest a sensing distance for the patches, and the comparison between each patch was performed at different frequency ranges, showing that each patch had a different sensitive frequency range. 

Yang, Hu [1] conducted a study on a two-story RC frame in which the damage scenario was applied by a shaking table. The authors proposed a sensitivity range of 0.40–0.60 m when using the frequency range of 60–100 kHz to calculate damage metrics. In terms of localized damage/cracks, Wang, Song [22] used three PZT patches which were distributed at different distances on the surface of a 2 m plain concrete beam. The PZT patch distribution was based on assuming the sensitive detecting distance was in the range of 0.40 m. The damage was induced, in this case, by initiating 5 localized 3 mm wide cracks ranging from 10–28 mm depth at different locations. The damage was determined by estimating the cross-correlation coefficient (CC) from the admittance response of each patch in the frequency range of 60–100 kHz. An agreement between the experimental results and the numerical results in terms of the calculated CC was found, and the closer the damage was, the smaller the recorded CC was.

Yang and Divsholi [2], and Divsholi and Yang [23] showed that using a narrow frequency range to estimate one RMSD value for damage quantification can give misleading results. In their study, a wide variation in the calculated RMSD values was noticed at different frequency ranges, and estimation of the RMSD values through sub-frequency intervals of 10 kHz has been suggested, as low-frequency RMSD was found to be sensitive to damage at a distance range of 300 mm from the PZT patch, whereas high frequency RMSD values were more sensitive to close distance damage [2].

In an attempt to increase the sensitivity of the EMI technique to damage, some studies extracted the SMI (see Equation (3)) from the admittance response in order to eliminate the effect of the mechanical impedance of the PZT patch [16,24,25]. By using embedded PZT patches and proposing a two-dimensional model to account for the thickness actuation in addition to the already accounted extensional actuation, Ai and Zhu [26] calculated and compared the RMSD values from the raw admittance signature, SMI, and the effective structural impedance (ESMI) of an RC beam. The authors concluded that the 2D model, which was used to extract the ESMI, rendered this parameter more accurate in quantifying the damage when compared with the raw admittance and the SMI-based RMSD. However, the authors also stated that all the mentioned parameters are sensitive to damage, and the sensing distance for the embedded PZT transducers was found to be 850 mm. 

Other studies have explored the ability of the EMI technique to monitoring long-term processes that affect the durability and strength gain of cementitious materials. During the early hydration period of mortar samples, the first 24 h after mixing with water, the resonance frequencies and amplitudes of the admittance signature have been shown to be affected by both the setting and the hardening processes. This is in an agreement with the results obtained from both the pin penetration resistance test, and the increase in the elastic modulus [27]. After 24 h, the same increase in the resonance frequency over time as well as the decrease in the amplitude were observed and related to the increase in the samples’ compressive strength [28,29]. With regard to EMI-durability-related studies, Talakokula et al. [30,31] used the EMI technique to monitor both the carbonation-induced and chloride-induced corrosion in RC samples. In these both mentioned studies, the PZT patch was attached to a rebar, which, in turn, was embedded into a concrete sample. Throughout the 230 day monitoring period for the carbonation-induced experiment, the equivalent stiffness parameter (ESP), which was extracted from the admittance signature through a suggested 2D model [32,33], showed two different stages. An initial stage was related to the increase in the matrix stiffness due to the blockage of the pores by calcium carbonate. This was before the decrease in ESP which was related to corrosion-attributed micro cracks and their effect on the material stiffness. Regarding the chloride-induced corrosion, the stiffness parameter and the mass parameter, which were extracted from the admittance signature, showed a decreasing trend, which is in compliance with the chloride exposure time. In addition, Liu et al. [34] assessed the effect of freezing–thawing on the EMI response, and it was shown that both the RMSD and the electrical resistance signature were sensitive to the process due to the change in the stiffness through the consecutive freeze and thaw cycles.

### 1.3. Research Significance

Following the above-mentioned studies, there is still an urge to address the sensitivity range of the EMI technique in terms of damage detection, especially when changes in the material properties take place due to increasing damage, which consequently affects the sensitivity of the technique. Therefore, in the first part of this paper, a detailed experimental program is followed to assess the damage distance and damage increase effect on the EMI response. 

On the other hand, and due to the lack of the studies that have addressed the effect of damage repair on the EMI, the second part is dedicated to addressing the effect of repair on the EMI response. This can facilitate the lending of the technique to onsite repair monitoring systems in cementitious structures.

## 2. Materials and Methods 

The experimental program used in this study was divided into a first part to assess the effect of damage on the PZT EMI response and a second part to assess the effect of repairing on the PZT EMI response. 

Two identical mortar beams of 100 mm × 100 mm × 500 mm were used throughout the experimental program. One beam was used in the damage experiment and the other on the repair experiment. Using two identical samples for the two different experiments provided the ability to check the repeatability of the damage effect on the EMI response, as in both experiments, damage was initiated first before either starting to increase the damage or repairing it. The binder used was CEM I 52.5N and the sand was siliceous CEN Standard sand with a grain size distribution ranging from 0.08 to 2.00 mm, conforming to BS EN 196-1 [35]. Mortars were cast with a sand/cement ratio of 3.0 and a water/cement ratio of 0.65. Mixing was carried out using a mechanical pan mixer of 0.1 m^3^ according to ASTM C192/C192M [36]. After mixing, the fresh mortar was poured into an oiled wooden framework in three equally thick consecutive layers. Each layer was vibrated for 2 min using an external vibrator before placing the next layer. After casting, the surfaces of the samples were covered with cling film to prevent evaporation. At 24 h after casting, the samples were demolded and cured in a curing room of 21 °C and 50% RH. The samples remained in the curing room for a minimum of 90 days before testing to ensure sufficient hydration.

**Figure 2:**
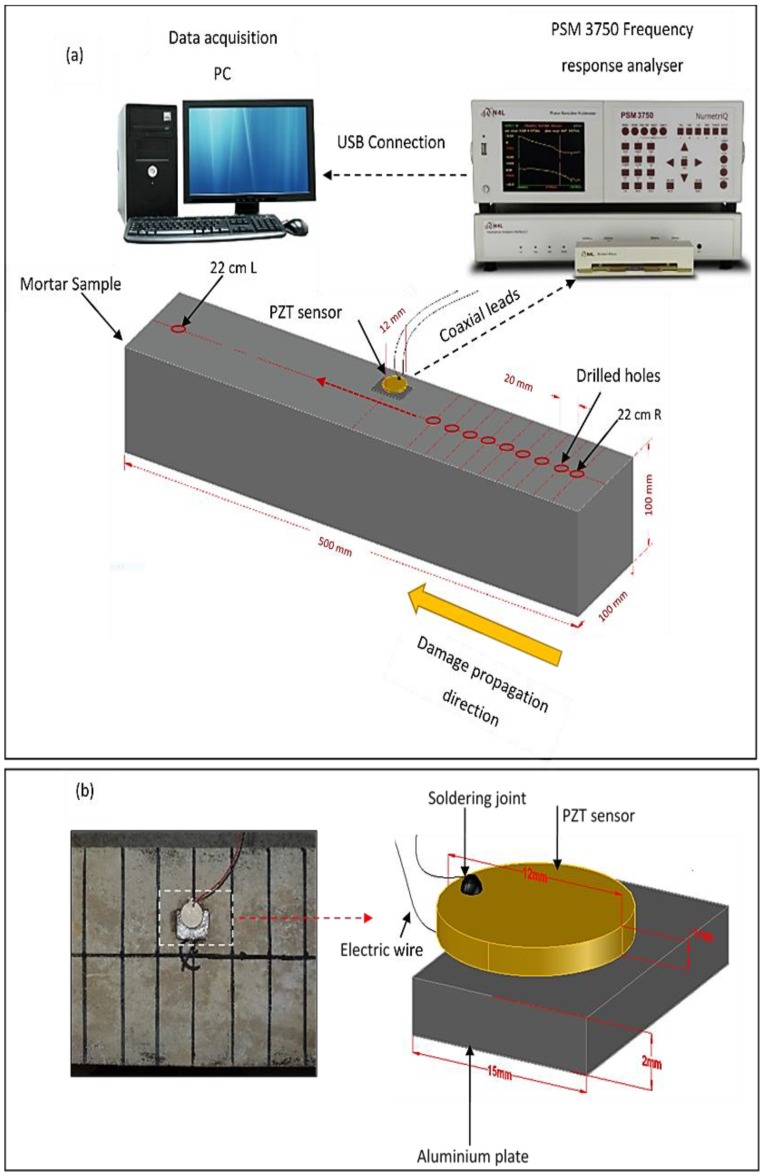
Schematic diagram showing the experimental setup for the electromechanical impedance technique (EMI) experiment: (**a**) Location of drilled holes in relation to PZT (**b**) PZT set-up.

**Figure 3:**
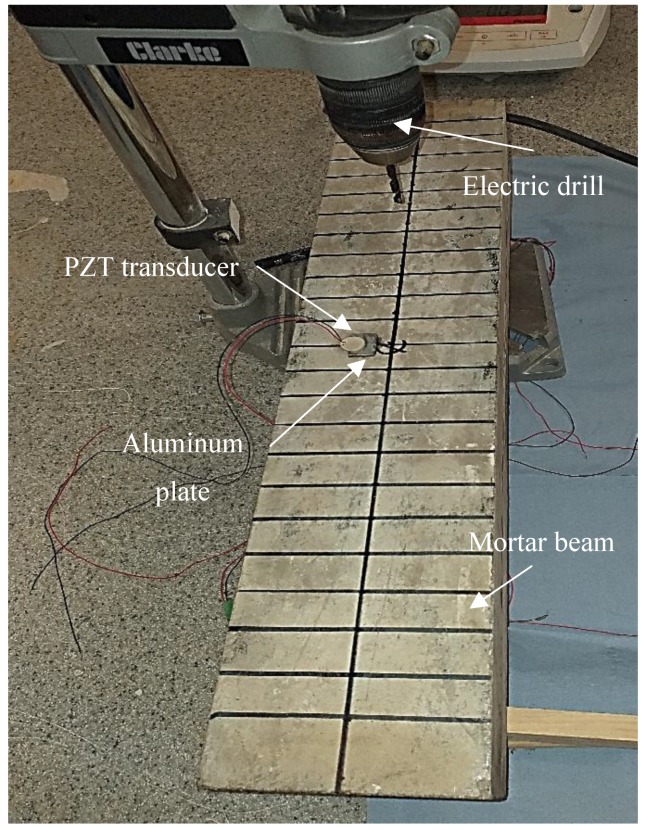
Mortar beam with the lead zirconium titanate (PZT) transducer attached to it.

The PZT setup used to acquire the EMI response is shown in Figure 2 and Figure 3. The setup was composed of a soft PZT patch produced at Thorlabs Inc (Ely, UK), which conformed with the THP51 PZT material type. The specifications of the PZT patch are shown in Table 1. The PZT patch was attached to an aluminum plate of 15 mm × 10 mm × 2 mm weighing 0.8 g. The aluminum plate was then attached to the surface of the mortar beam with a 10 mm offset from the beam’s centerline (see Figure 2a). A Cyanoacrylate CN-Y adhesive type from Tokyo measuring instruments labs (Tokyo, Japan) was used to attach the PZT patch to the aluminum plate as well as the aluminum plate to the beam. The purpose of using this setup was to decrease the effect of any possible local heterogeneity in the interface between the PZT and the mortar, which might affect the efficiency of the mechanical coupling. The measurement of the setup was commenced after at least 24 h to ensure the bonding agent was sufficiently cured so as not to affect the measurements. 

The PZT patch was connected through 50 Ω coaxial cables to a Newtons4th PSM 3750 frequency analyzer (Newtons4th Ltd., Leicester, UK) interfaced with an impedance analyzer (see Figure 2a). The frequency analyzer was controlled by a PC which was connected through a USB cable. The voltage amplitude used to collect the admittance response was 1 V, and the frequency range used was 15 to 350 kHz. Through this frequency range, 1000 points were collected at 335 Hz intervals.

### 2.1. Damage Stage Experiment

In this experiment, the mortar beam was subjected to five different damage stages (see Table 2 and Figure 2a). 

In each damage stage, 22 holes were drilled by an electric drill on the surface of the beam. The drilling started with hole number 1 at a distance of 220 mm away from the center of the beam, denoted as 22 cm R in Figure 2a and along the center line of the beam. Additional holes were subsequently drilled in a line approaching and passing the center of the beam at 20 mm intervals which ended with hole number 22 and is denoted as 22 cm L (see Figure 2a).

Hole diameters of 4, 5, 6, 8, and 10 mm were used for damage stages 1, 2, 3, 4, and 5, respectively (see Table 2). The admittance response for the beam was collected before and after each hole was drilled to assess the effect of the increase in damage and the effect of distance on the admittance response.

### 2.2. Repair Stage Experiment

An identical beam was used to investigate the effect of damage repair on the EMI response. Damage was simulated by drilling 16 holes of 10 mm diameter and 5 mm depth at 15 mm spacing along the center line of the beam. The repair was simulated by filling the drilled holes with a cement paste (CEM 1 52.5N, water/cement ratio of 0.35). The admittance signature was obtained before and after drilling each hole. After filling the holes with the cement paste, the admittance signature was collected for a period of 60 h to assess the repair effect on the EMI response. In order to minimize the effects of the ambient temperature and relative humidity, the admittance signatures for the sample were collected before and during the repair process, while the sample was in an environmental chamber of 21.8 °C and 68% RH. These two values were specifically selected as they represent the average laboratory temperature and relative humidity during the drilling.

In order to assess the temperature variations during the repair process, a K type thermocouple was attached to the center of the sample adjacent to the PZT patch. The thermocouple wires were connected to a PC-controlled TC-08 data logger (Pico Technology, St Neots, UK).

## 3. Results and Discussion

### 3.1. Increasing Damage Effect

**Figure 4:**
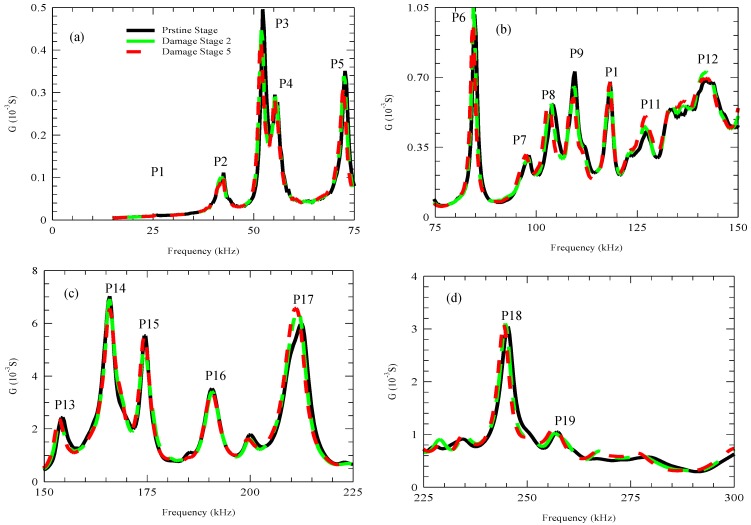
Admittance signature for the pristine stage, damage stage 2, and damage stage 5 at the frequency ranges of (**a**) 15–75 kHz, (**b**) 75–150 kHz, (**c**) 150–225 kHz and (**d**) 225–300 kHz.

Figure 4 shows the real part of the admittance signatures for three representative damage stages: the pristine stage, damage stage 2, and damage stage 5. 

**Figure 5:**
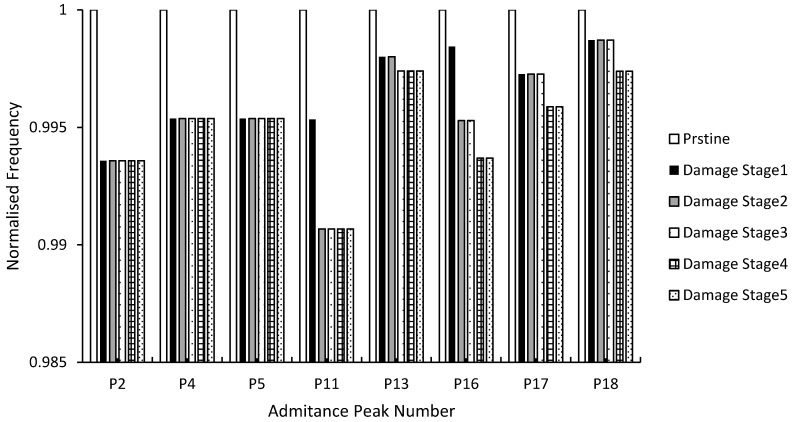
Normalized frequency for the admittance signature’s peaks.

In the frequency range investigated, several resonance peaks were observed, and the maximum resonance peak amplitude recorded was 7.06 × 10^−3^ S. This maximum value was recorded for the pristine stage, at 165.9 kHz, which is denoted as peak P13. Damage stage 2 and damage stage 5 recorded lower amplitude values of 6.91 × 10^−3^ S and 6.55 × 10^−3^ S, respectively, for peak P13. At the higher frequency ranges—P16, P17, and P18—it was noticeable that the real part of the admittance signature increased as the damage level in the sample increased. For instance, the pristine stage at peak P16 recorded a value of 5.99 × 10^−3^ S; however, both damage stages 2 and 5 recorded higher values of 6.48 × 10^−3^ S and 6.57 × 10^−3^ S, respectively. From peak number P16, it was noticeable that as the damage level in the sample increased, a frequency shift towards lower frequencies (left-hand side) took place. Therefore, P16 at the pristine stage occurred at relatively higher frequencies than the other two damage stages (see Figure 4). This frequency shift also occurred on the other resonance peaks, as shown in Figure 5.

Figure 5 shows selected admittance peak frequencies, which were normalized by their pristine stage frequencies. Figure 5 highlights the frequency shift through the different damage stages. It was evident that higher frequencies within the frequency range used were more sensitive to detecting increasing damage than lower frequencies [37]. Even though peak numbers P2 to P5 clearly showed a higher resonance frequency for the pristine stage, damage stage 1 to damage stage 5 showed equal resonance frequency values, regardless of the increasing damage. On the other hand, by considering peak numbers P13 to P18, which were at frequencies higher than 150 kHz, the resonance frequency became more sensitive to increasing damage; this manifested itself through the decreasing trend of P13 and P16 frequencies as the damage level in the sample increased.

The inverse trend between the resonance frequencies and the admittance amplitude was observed during the hydration and hardening in the first 28 days. During these periods, the admittance amplitude showed a decrease over time, and the resonance frequency showed an increase, indicating an increase in the material stiffness [16,27,29]. Therefore, the decrease in the resonance frequency and the increase in the admittance amplitude indicated a decrease in the material stiffness in the vicinity of the PZT as well as an increase in the damping properties of the material [28,38]. 

**Figure 6:**
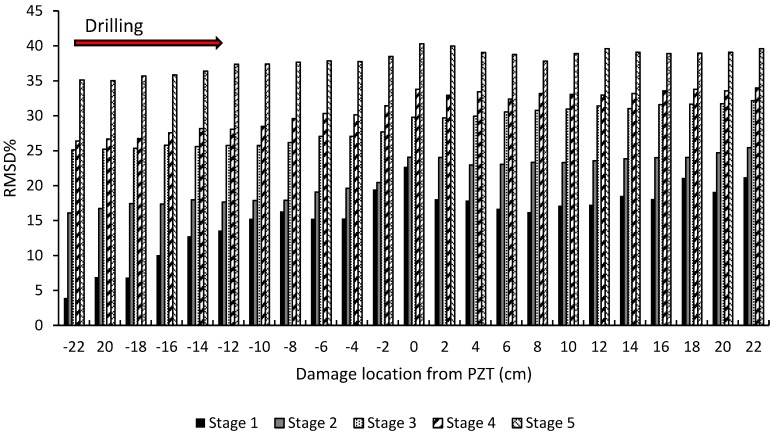
Root-mean-square deviation for damage stages 1, 2, 3, 4, and 5, as a function of distance from the PZT sensor.

The RMSD was used in this study to quantify the changes in the admittance signature through the different damage stages. Figure 6 shows the RMSD through the different damage stages relative to the initial pristine stage. As shown in Figure 6, the PZT sensor was able to sense an increase in damage up to as far as 220 mm away from the PZT location. By taking the first hole, which is denoted as −22 cm, the RMSD for damage stage 1 at this location recorded a value of 3.93%; however, for damage stages 2, 3, 4, and 5, it recorded values of 16.10%, 25.11%, 26.36%, and 35.12%, respectively, which showed a clear increasing trend as the damage level increased. A similar increasing trend was observed at the other damage locations. However, it should be noted that the increase in the damage sensed at the different positions shown in Figure 6 was due to both the previous damage stage and the current damage stage. Also, in Figure 6, as the damage approached the PZT sensor, an increase in the RMSD was observed. By comparing the value of the RMSD for stage 1 at damage locations −220 mm and 220 mm, the values of the RMSD recorded 16.10% and 21.21%, respectively, which showed an increase due to the increase in the damage level in the beam.

### 3.2. Damage Location Effect

**Figure 7:**
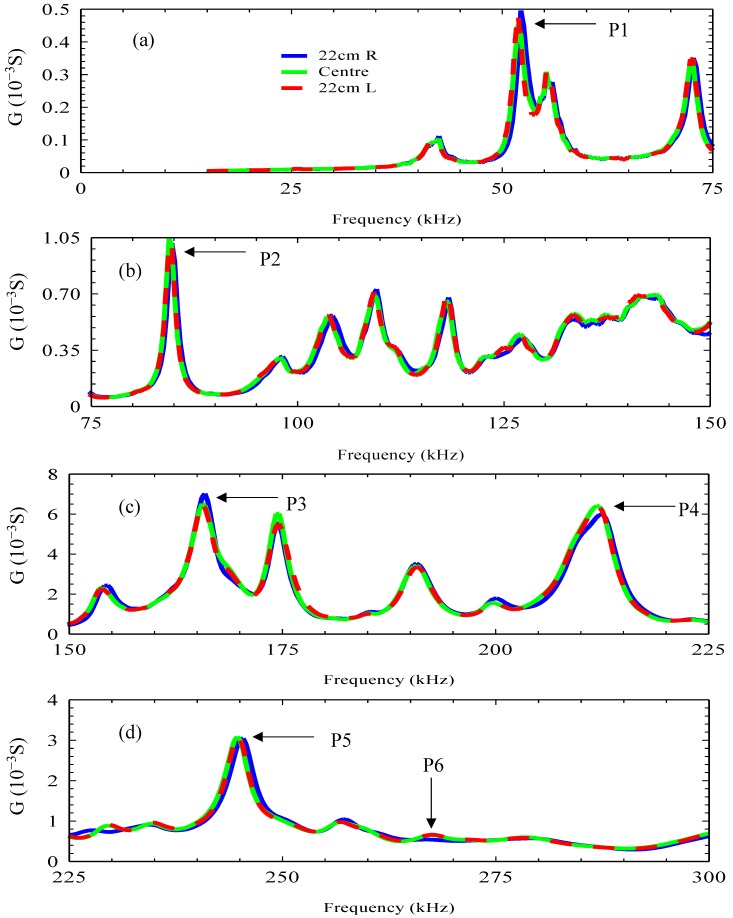
Admittance signature at distances of 22 cm left and right from the location of the PZT sensor and at the center of the beam at frequency ranges of (**a**) 15–75 kHz, (**b**) 75–150 kHz, (**c**) 150–225 kHz and (**d**) 225–300 kHz

To investigate the effect of damage approaching and receding from the PZT on the EMI response, Figure 7 shows the real part admittance signature for damage stage 1 at the following locations:

1. Hole 1 which is 220 mm to the right of the PZT sensor, denoted 22 cm R, which was the starting point of damage propagation;
2. The center of the beam, which is the location of the PZT sensor;
3. At 220 mm to the left of the PZT sensor, denoted 22 cm L, which is the last drilled hole in the beam.

By comparing these three signatures, it was observed that the 22 cm R signature’s resonance peaks took place at higher frequencies than the center signature and the 22 cm L signature. For instance, P1 at the 22 cm R signature took place at the frequency of 52.22 kHz, and both the center and the 22 cm L signatures’ P1s took place at 51.887 kHz. This decrease in the frequency of resonance peaks as damage propagated from the right to the left of the sensor was due to the decrease in the stiffness of the material and the increase in previously described damage.

It is interesting to note that both the center signature and the 22 cm L signature were almost similar and followed the same trend, which was different to that observed at 22 cm R where there was a different signature trend. This can be clearly seen by comparing the three signatures within the frequency range of 265 to 275 kHz shown in Figure 7d; the center and the 22 cm L signatures showed an additional resonance peak, which is denoted as P6, which did not appear on the signature of the 22 cm R.

The behavior of the three admittance signatures shown in Figure 7 suggests that as the damage propagated towards the PZT transducer, the admittance signature sensitivity to damage increased. However, as the damage moved away from the PZT transducer, the sensitivity to damage decreased. 

**Figure 8:**
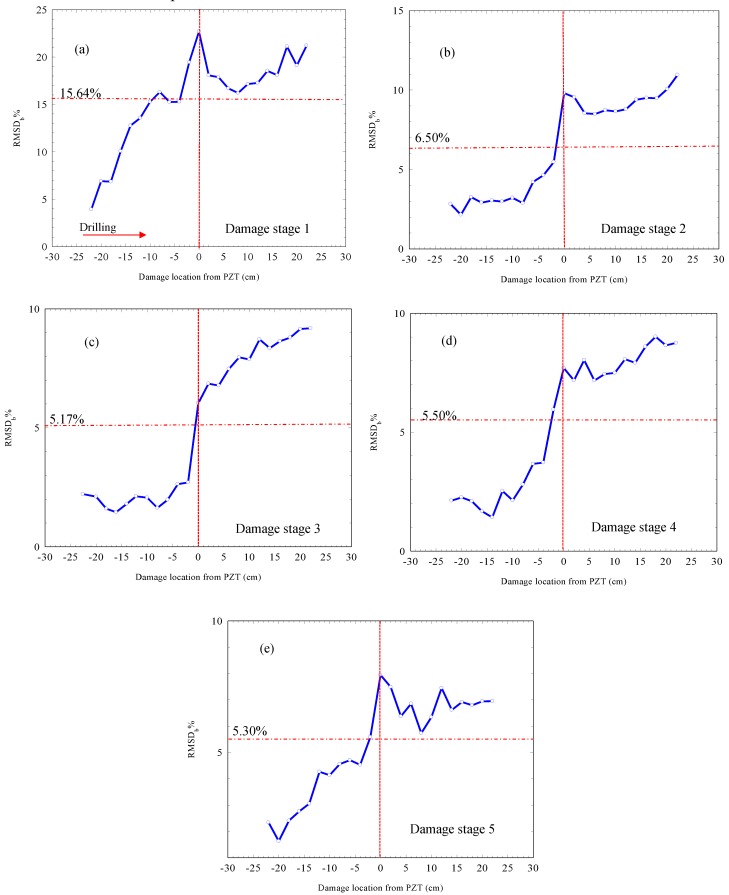
RMSD_b_% calculated after each drilled hole for (**a**) damage stage 1, (**b**) damage stage 2, (**c**) damage stage 3, (**d**) damage stage 4, (**e**) and damage stage 5.

In order to investigate the PZT sensitivity to damage propagation, the RMSD_b_ was investigated. In the case of RMSD_b_, the “pristine stage” was taken as the stage before drilling the first hole in each damage stage, rather than the “absolute pristine stage” as was the case for the RMSD calculation in Figure 6. This alternative approach allowed any effect of an increase in damage on the RMSD_b_, due to the damage caused within the same damage stage, to be identified and not the cumulative damage from previous damage stages. Figure 8 shows the RMSD_b_ at different damage locations relative to the PZT sensor location.

The following observations can be noted from Figure 8. First, a continuous increasing trend for the RMSD_b_ was apparent as the damage propagated from the right-hand-side of the beam (negative direction) to the left-hand-side of the beam (positive direction). This continuous increase in the RMSD_b_ once again proves the sensitivity of the admittance signature to increasing damage. Secondly, the RMSD_b_ for damage stage 1 showed a higher rate of increase when compared with the other damage stages. This was attributed to the formation of a new hole at this stage as opposed to the widening of the hole which took place in the later stages. Therefore, the RMSD_b_ at stage 1 increased from approximately 5% to more than 20%; however, for the other damage stages, this parameter increased from 5% to less than 10%. Thirdly, the increasing rate for the RMSD_b_ started to decrease as the damage receded from the location of the PZT. Fourth, the RMSD_b_ decreased as the damage stage increased, in contrast to what was observed in Figure 6. This was evident as the average value of the RMSD_b_ at damage stage 1 recorded a value of 15.64%, which decreased to 6.5% at damage stage 2, until reaching 5.30% at damage stage 5.

**Figure 9:**
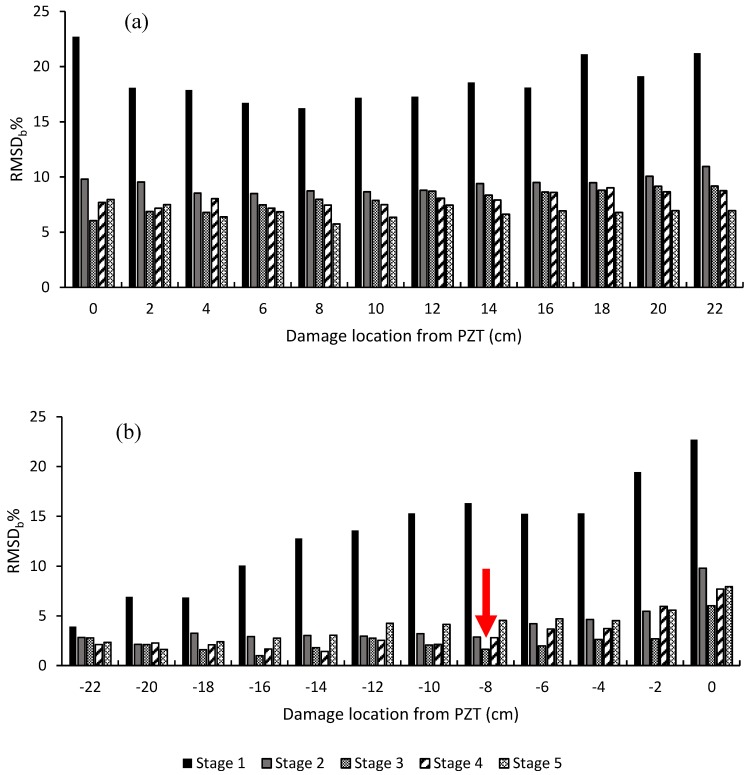
Root-mean-square deviation using the pre-damage stage for each stage as a pristine stage, RMSD_b_, for (**a**) the damage receding from the PZT and (**b**) the damage approaching the PZT.

The last two points suggested that the sensitivity of the PZT to damage was affected by the location of damage relative to the PZT as well as the damage level in the sample. This can be seen more clearly in Figure 9 where the RMSD_b_ values for the different stages at the location of each hole are compared. 

It can be observed that damage stage 1 recorded the highest RMSD_b_ values through all the damage locations due to the high damage induced at this stage, as described previously. For the other damage stages, and as the damage receded from the PZT location (see Figure 9b the positive side of the beam), a decreasing trend was witnessed on the RMSD_b_ values as the damage stage increased. This was evident 40 mm away from the PZT location and through all the subsequent positions, as damage stage 1 recorded a higher RMSD_b_ than damage stages 2, 3, 4, and 5 respectively.

On the right-hand-side of the beam (negative side), as shown in Figure 9a, it was noticed that the RMSD_b_ followed two general behaviors:

(a) The RMSD_b_ decreased as the damage level increased in the sample through damage stages 1, 2, and 3.
(b) Then, after damage stage 3, the RMSD_b_ started to increase again through damage stages 4 and 5, as shown by the red arrow in Figure 9b, at the −8 cm position. This was particularly evident as the damage approached the PZT from a distance of 80 mm. This behavior continued until 20 mm to the left side of the PZT, the positive side of the beam.

These later different behaviors of the RMSD_b_ can be related to the behavior of the mechanical elastic waves which are transmitted and reflected from the edges of the holes back to the PZT sensor. As the damage approached the PZT, the waves generated from the PZT were efficiently transmitted and reflected back to the PZT through the less damped material between the PZT and the newly formed damage. Therefore, as the damage approached the PZT from the right-hand-side, the PZT became more sensitive to damage and was sensitive to distinguishing small changes in diameter between damage stage 3 and damage stage 4. However, as the damage increased in the material and receded from the PZT at the same time, the damping effect of the material became more significant. This was due to the increase in the number of holes between the new damage and the PZT; hence, the damping of the material increased and the transmitted waves lost their energy more rapidly, hence decreasing the admittance response sensitivity to damage. Therefore, a reduction in the RMSD_b_ took place with an increasing damage distance from the PZT.

These results indicated that the sensitivity of the PZT sensor to damage were dependent on the health condition of the mortar beam, as increasing damage in the material affected the EMI sensitivity to detect further damage due to the increase in wave energy dissipation.

### 3.3. Effect of Repair on the EMI Response

In this section, the effect of repair on the EMI response of the surface attached PZT is investigated.

**Figure 10:**
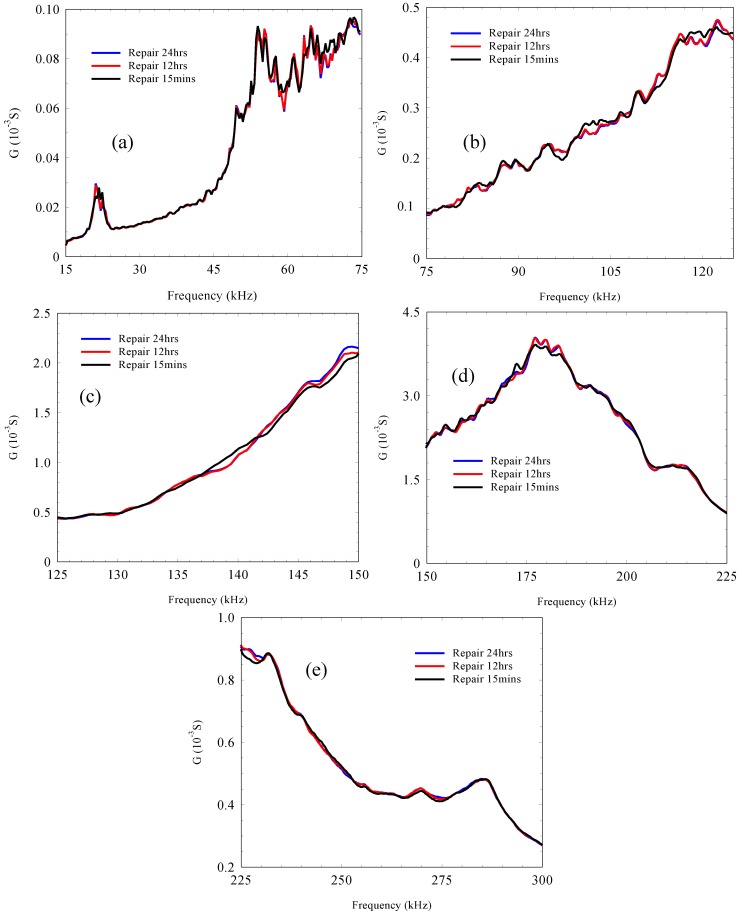
Admittance signature for the PZT transducer at 15 min, 1 h, and 24 h after repair in a controlled environment at frequencies of (**a**) 15–75 kHz, (**b**) 75–125 kHz, (**c**) 125–150 kHz, (**d**) 150–225 kHz and (**e**) 225–300 kHz.

Figure 10 shows the admittance signature through the whole frequency range for the PZT transducer at 15 min, 12 h, and 24 h after the holes were filled with cement paste. 

Figure 10a–d show a similarity between the 12 h repair and the 24 h repair admittance signatures. This was evident when assessing the admittance signature features within the frequency range of 60–75 kHz, as the anti-resonance peak, which was centered at around 60 kHz and showed a higher amplitude of 6.63 × 10^−5^ S for the 15 min repair signature as opposed to 6.25 × 10^−5^ S for both the 12 h and the 24 h admittance signatures respectively. 

Despite the noticeable difference between the 15 min repair admittance signature and the 12 h and 24 h admittance signatures, there was no general trend throughout the whole frequency range which could be used to describe this difference. By considering the frequency range of 75–125 kHz, both the 12 h and 24 h admittance signatures recorded higher amplitude values compared with the 15 min admittance signature, particularly at 117 kHz. On the other hand, lower values for the former were recorded at 120 kHz. With regard to the highest resonance peak, which was centered at 177 kHz, both admittance signature at 12 h and 24 h recorded noticeably higher amplitudes of 4.03 × 10^−3^ S and 4.04 × 10^−3^ S, respectively, when compared with the 15 min admittance signature, which recorded a value of 3.91 × 10^−3^ S.

The differences between the early admittance signature following repair, which is represented by the 15 min signature, and the later admittance signature following repair, which is represented by both the 12 h and the 24 h admittance signatures, allows the following key points to be drawn:

(a) The repair process caused a noticeable difference in the admittance signature with time. This was due to the hydration of the paste used for the repair and, hence, a change in the host material properties, such as damping and stiffness properties.
(b) The similarity between the 12 h and 24 h admittance signatures and the difference between these and the 15 min admittance signature suggest that most of the effect of the hydration of the repair paste and its effects on the EMI took place in the 12 h period initially after filling the holes with cement paste.
(c) The repair process affects the various frequency ranges differently, as some frequency ranges showed a decrease in the admittance signature amplitude, whereas other frequency ranges showed an increase in the admittance signature amplitude as the repair process proceeded. This indicates that the repair methodology employed in this experiment did not restore the specimen to its condition before damage was introduced.

**Figure 11:**
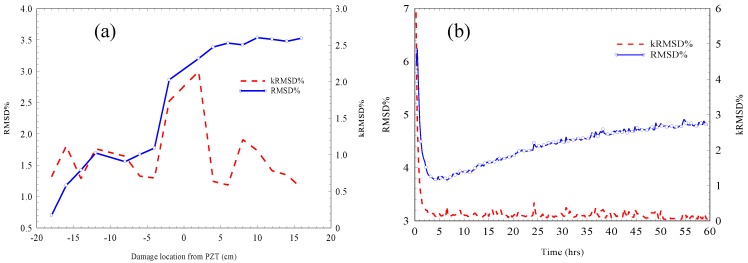
RMSD and kRMSD during (**a**) the damage process and (**b**) the repair process.

In order to assess the effects of damage and repair quantitatively, the RMSD and the relative RMSD (kRMSD) [24] were both calculated within the frequency range of 75–90 kHz which was the same frequency range in which the RMSD was calculated in the first damage experiment described in the damage experiment section. Figure 11 shows the RMSD and the kRMSD values through both the damage process and the first 60 h after filling the holes with cement paste. 

The pristine stages for the RMSD in the damage and the repair processes shown in Figure 11 were the first admittance signatures obtained at the beginning of each process. It is apparent from Figure 11a that the RMSD exhibited a continuous increase as more holes were drilled; hence, it was affected by the increase in damage. Regarding the kRMSD, it is evident that at a 200 mm distance from the PZT transducer, it recorded a value as low as 0.5%; however, as the damage approached the sensor location, the kRMSD increased rapidly to record a value of almost 2%. This increase in the kRMSD was also reflected by the increasing rate of RMSD as the damage approached the sensor location. Therefore, it was evident that the 75–90 kHz frequency range was sensitive to damage, which suggests that this frequency range is repeatable for the PZT setup used in this study. This has significant implications for the elimination of the trial and error processes which are normally necessary in the EMI damage detection method when finding a sensitive frequency range to estimate the damage metrics.

With regard to the repair effect on the RMSD and the kRMSD, and by considering Figure 11b, it is interesting to note that both these parameters started at high values which then decreased rapidly over time. This is in contrast to the effect of increasing damage, which showed an increase in the RMSD (see Figure 11a). This decreasing trend in both the RMSD and the kRMSD continued until almost 5 h after filling the holes with cement paste. Thereafter, the RMSD followed a gradual increasing trend, which continued until the end of the experiment at 60 h. On the other hand, the kRMSD followed a constant stable trend recording values in the range of 0.2% until the end of the monitoring period. 

These trends for the RMSD and the kRMSD suggest that two different processes took place during the repair. The first process took place at the start of the measurements until the age of 5 h due to the decrease in the RMSD, and the second one started to intervene thereafter due to the increase in the RMSD. The decrease in the RMSD indicates that the first process, which contributed to the high initial RMSD value, was withdrawn or decreased over the first few hours; therefore, the RMSD decreased, approaching low values in this case. The second process effect on the RMSD suggested that this process introduced changes to the material properties; therefore, an increase in the RMSD took place similarly to in the damage process case, albeit at a slower rate compared with the first process.

**Figure 12:**
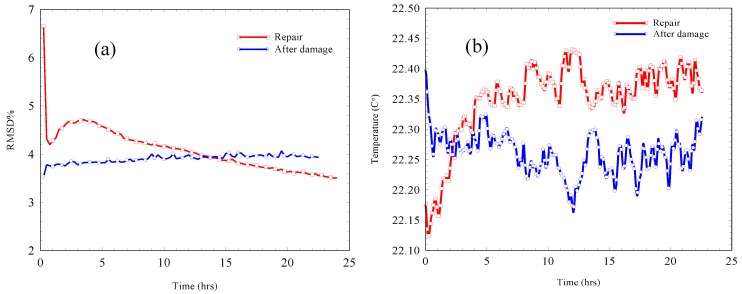
After damage and during repair responses for (**a**) RMSD% and (**b**) temperature.

In order to allow a comparison between the RMSD after damage and during repair, Figure 12 shows the RMSD for both the 24 h period after damage, i.e., after drilling all the holes, and the first 24 h period throughout the repair process. The pristine stage used in Figure 12 for both the after damage and the repair RMSD was the admittance signature before initiating the damage on the sample, i.e., the admittance signature before drilling hole number 1 in the repair experiment. Considering the RMSD through the repairing period in Figure 12a, it is interesting to note that the RMSD exhibited different trends when compared with the RMSD for the same period in Figure 11. The RMSD in this case, Figure 12a, showed a sudden drop during the first hour; however, thereafter, it showed a gradual increase until it levelled out after 3 h. At this point, it then began to decrease until the end of the 24 h period. In Figure 11, the decrease in the RMSD continued until almost 5 h, and then, in contrast to the trend of the RMSD in Figure 12, the RMSD started to increase gradually. These different trends observed for the RMSD in the two figures were due to the difference in the pristine stage used in each case.

By considering the temperature response before and during the repair process, which is shown in Figure 12b, temperatures following damage throughout the monitoring period fluctuated between 22.2 and 22.4 °C with an average value of 22.3 °C. During the repair process, the temperature fluctuated between 22.1 and 2.4 °C with an average value of 22.4 °C throughout the 24 h monitoring period.

By comparing the temperature response and the RMSD response during the repair process, both the parameters mentioned showed a quick drop during the first hour, and then an increasing trend took place. These two trends observed in the temperature response could be attributed to delays in the time taken for the samples to reach equilibrium within the environmental chamber, as the repair processes was undertaken under ambient laboratory conditions, and the temperature increase was attributed to an ongoing exothermic hydration reaction during the cement paste repair.

Therefore, taking into consideration the time needed for filling the holes outside the environmental chamber, which was approximately 1 h after mixing the cement with water, the increase in the temperature shown during the repair period could be related to the end of the cement induction period and the beginning of the acceleration stage. Therefore, both the rapid decrease and the gradual increase in the RMSD until an age of 3 h can be attributed to the change in the sample temperature due to reaching equilibrium as well as the hydration reaction of the filling cement paste.

After the 5 h repair period, and despite the relatively stable temperature, the RMSD started to decrease, which was opposite to its behavior throughout the prior stage of repair. By 15 h after the repair process, the repaired sample started to show lower RMSD values compared to those after the damage. By the age of 60 h, the RMSD for the after-repair process recorded a value of 3.27% in comparison to 3.93% for the after-damage stage. This decreasing trend for the RMSD over time indicated that the effect of the repair on the material properties started to dominate after 15 h from cement paste hydration; hence, the RMSD started to decrease. The decrease in the RMSD suggested that a partial recovery of the material properties took place. This recovery is predicted to be due to an increase in the mass of the material; however, no appreciable recovery in the material stiffness was expected to take place, as the admittance signature response did not show an observable right-hand-side resonance frequency peak shift.

## 4. Conclusions

This study was conducted to assess the effects of damage and repair on mortar beams through the electromechanical response of a surface-attached PZT transducer. The RMSD in this study was calculated through the frequency range of 75–90 kHz, as this frequency range has shown to be sensitive and repeatable in detecting both the damage and the effect of repair on the configuration used. Overall, it was shown that damage could be detected 200 mm away from the PZT transducer depending on the damage intensity with greater sensitivities within 100 mm of the PZT transducer. Furthermore, for the first time, it was shown that admittance signatures could detect a recovery in performance as a result of repair. Consequently, this system shows great promise in sensing the damage and self-healing of mortar beams. 

More specific conclusions are as follows:

(a) During the damage experiment:
  1. By assessing the resonance frequency amplitudes, it was shown that increased damage decreased the resonance frequency and increased the admittance amplitude, which indicates a decrease in the material stiffness as well as an increase in the damping properties of the material.
  2. By using the RMSD as a damage metric, it was shown that as the damage level increased in the material, the RMSD also followed an increasing trend.
  3. Using different pristine stages for RMSD estimation enabled the detection of both accumulated damage and the effect of damping on the PZT sensitivity to damage.
  4. The PZT setup used in this study was more sensitive to damage approaching the sensor than that receding away from it. This was attributed to the efficient reflection of the vibrational waves from the approaching damage in contrary to the case of the receding damage in which the waves were dissipated by the presence of existing damage.
(b) During repair:
  1. Changes in the admittance signature were detected when the stage following damage was compared to that after repair. However, no single general trend was detected, as each frequency range showed uniquely changing features. This was attributed to the inability of the repair methodology to restore the specimen to its original state.
  2. The sensitivity of the RMSD to the temperature of the fused cement passed up to 5 h following repair and was related to the acceleration period of the hydration reaction.
  3. Fifteen hours after filling the holes with cement paste, the RMSD showed a clear decreasing trend over time which was in contrast to its behavior before the repair processes. This decreasing behavior was attributed to the partial recovery of the material properties and, in particular, to the decrease in the damping properties and the increase in the mass. However, the admittance signature did not indicate a gain in stiffness.

## Figures and Tables

**Table 1:** Physical Properties of the PZT material.

Property	Value
Density (g/cm^3^)	10
Electric Permittivity, $\varepsilon_{33}^{T}$/$\varepsilon_{0}$	3300
Piezoelectric Strain Coefficient, $d_{31}$(10^−12^ C/N)	−320
Piezoelectric Strain Coefficient, $d_{33}$(10^−12^ C/N)	710
Elastic Compliance Coefficient, $S_{11}^{E}$(10^−12^ m^2^/N)	17
Elastic Compliance Coefficient, $S_{33}^{E}$(10^−12^ m^2^/N)	23
Dielectric Loss Factor, tanδ (10^−3^)	2.2
Mechanical quality factor (Q_m_)	60

**Table 2:** Hole diameters used for damage stages 1 to 5.

Damage Stage Number	Diameter of Holes (mm)	Accumulative Extracted Volume at Each Stage (mm^3^)
Stage 1	4	1382
Stage 2	5	2159
Stage3	6	3109
Stage4	8	5526
Stage5	10	8635
